# Pathological PCI as a prognostic marker of survival after neoadjuvant chemotherapy in patients undergoing interval cytoreduction with or without HIPEC in FIGO stage IIIC high grade serous ovarian cancer

**DOI:** 10.3389/fonc.2024.1458019

**Published:** 2024-08-20

**Authors:** Snita Sinukumar, Dileep Damodaran, Deepika S., Sanjay Piplani

**Affiliations:** ^1^ Department of Surgical Oncology, Jehangir Hospital, Pune, India; ^2^ Department of Surgical Oncology, MVR Cancer Centre and Research Institute, Calicut, India

**Keywords:** pathological PCI, interval cytoreductive surgery, high-grade serous ovarian cancer, HIPEC, KELIM

## Abstract

**Objective:**

To determine the best possible value of pathological PCI (pPCI) as a prognostic marker for survival in high-grade serous epithelial ovarian cancer patients in patients treated with neoadjuvant chemotherapy and interval cytoreductive surgery.

**Methods:**

All patients with FIGO stage IIIC high-grade serous ovarian carcinoma were included. Receiver operating curves (ROC) were used to determine the best possible score for pPCI in predicting survival. Survival curves were calculated using the Kaplan–Meier test, and factors affecting survival were compared using the log-rank test.

**Results:**

From January 2018 to January 2024, 171 patients who underwent interval cytoreductive surgery were included. Complete cytoreduction was achieved in 88% of the patients. ROC curves determined a (pPCI) cut-off value of 8 as the best possible score for predicting survival with a sensitivity of 82% and specificity of 67% (Youden’s Index = 0.60). pPCI with a cut-off value of 8 showed improved OS (p = 0.002) and DFS, (p = 0.001) in both univariate and multivariate analyses.

**Conclusion:**

Following interval cytoreductive surgery, despite optimal complete cytoreductive surgery, a pathological PCI of 8 is a poor prognostic indicator of survival and may serve as a surrogate clinical marker for guiding clinicians in adjuvant treatment, especially in resource-driven settings in the real world.

## Introduction

The use of neoadjuvant chemotherapy (NACT) for the treatment of advanced ovarian cancer is a widely accepted approach. The rationale for the use of this approach is mainly for those patients who cannot be subjected to complete primary surgical cytoreduction, either due to extensive disease spread or poor performance status that makes it less amenable to aggressive cytoreductive surgical treatment (1–3).

While optimal cytoreductive surgery remains the most important prognostic factor for patients with advanced ovarian cancer (3, 4) there are a limited number of studies that have identified prognostic factors post NACT at interval cytoreductive surgery.

For patients with peritoneal metastasis, the extent of disease involvement is assessed by Sugarbaker’s peritoneal carcinomatosis index (PCI). The peritoneal cancer index (PCI) assessed by the surgeon intraoperatively and also referred to as the surgical PCI (sPCI) is a well-established prognostic predictor in patients undergoing CRS for various cancers metastasizing to the peritoneal cavity (5–7). However, regarding interval cytoreduction, following a response to neoadjuvant chemotherapy (NACT), peritoneal deposits are often replaced by areas of scarring that could be potential sites for residual disease in nearly 30% of the patients (8, 9). It has been shown that areas harboring residual disease could have a benign appearance or appear normal and it may be prudent to resect previous areas of peritoneal involvement (8, 10, 11). In the context of interval cytoreductive surgery, visual inspection has its inherent fallacies and the surgical peritoneal carcinomatosis index (sPCI) is subjective and may overestimate or underestimate the extent of diseases in patients undergoing CRS after NACT. Furthermore, following neoadjuvant chemotherapy, studies have reported a concordance rate of only 21.2% between the surgical PCI and pathological PCI (12).

Therefore, in the context of interval cytoreduction, it is reasonable to assume that pathological PCI (pPCI) would place more clinical significance than surgical PCI. Surgical PCI can be used to assess surgical feasibility and probability of complete cytoreduction. It is unclear whether neoadjuvant chemotherapy can be used as a prognostic factor for survival. Pathological PCI (pPCI), on the other hand, can reflect the true peritoneal burden of the disease after neoadjuvant chemotherapy (NACT) and is probably better for determining patient prognosis than surgical PCI in this setting.

Furthermore, a cut-off point for pPCI to assess prognostic significance after interval cytoreductive surgery after NACT has not been identified, and to the best of our knowledge, no study has truly evaluated the prognostic role of pPCI following interval cytoreductive surgery in ovarian cancer.

In patients receiving NACT, the rate of CA-125 decline during chemotherapy treatment has been correlated with increased chemosensitivity and is reflected as the KELIM score. The higher the KELIM, the faster the CA-125 elimination, and the higher the chemosensitivity. The role of CA-125 ELIMination Rate Constant K (KELIM) as a marker of chemosensitivity in ovarian cancer patients and its prognostic and predictive significance after NACT has been established through various studies (13, 14). Most of these studies have used KELIM as a predictive factor for achieving optimal cytoreduction (15). The higher the KELIM score, the greater the chemosensitivity and therefore, the higher the probability for optimal cytoreduction. But is there a correlation between this chemosensitivity and the amount of residual disease resected at surgery? A high KELIM may indicate tumor chemosensitivity but may not always signify the absence or the amount of residual disease.

The aim of the study was therefore, to determine the best possible value of pathological PCI score as a prognostic marker for survival in patients with stage IIIC high grade serous epithelial ovarian cancer patients and to correlate the KELIM Score with the pathological PCI in patients treated with neoadjuvant chemotherapy and interval cytoreductive surgery, with or without HIPEC.

## Materials and methods

This is a retrospective analysis of prospectively collected data. Patients with advanced epithelial ovarian, fallopian tube, and primary peritoneal cancer (stage IIIC) who underwent interval CRS following NACT were included in the study. Before performing NACT, radiological PCI was calculated, and tissue diagnosis was obtained via transcutaneous abdominal biopsies performed by an interventional radiologist. Diagnostic laparoscopy adds value to pretreatment assessment before neoadjuvant chemotherapy. We did not subject the patient to laparotomy for PCI evaluation prior to starting NACT. PCI evaluation before starting NACT was radiological PCI evaluation, and a diagnosis of ovarian cancer was established by biopsy with the help of an interventional radiologist.

Patients under the age of 18 years, with non-serous high-grade histology who underwent upfront CRS, second look surgery, or did not undergo surgery after NACT were excluded from the study. The KELIM score was calculated using at least three CA-125 values within the first 100 days of chemotherapy, using a validated calculation formula [https://www.biomarker-kinetics.org/CA-125-neo] (16). The KELIM score was analyzed as both a continuous and binary variable, with a cut-off point of <1 or >1. Preferably the CA-125 values before cycles 2, 3, and 4 were used to calculate the KELIM score. Patients received doublet neoadjuvant chemotherapy with paclitaxel and cisplatin. None of the patients received neoadjuvant bevacizumab.

### Surgical intervention

All surgical procedures were performed with the goal of obtaining a complete cytoreduction (no visible residual disease). Briefly, a midline incision from the xiphoid to the pubis was employed regardless of the extent of the disease. The disease was quantified using Sugarbaker’s peritoneal cancer index (PCI) (6, 17). In this study, all scarred areas and suspicious areas of the peritoneum were resected along with grossly involved diseased areas, following a systematic and predefined protocolized template described elsewhere (18). This peritonectomy comprised the resection of peritoneal linings of the pelvic, antero-parietal, right and left upper quadrant, along with a total omentectomy (greater and lesser omentectomy). The completeness of cytoreduction was reported using the completeness of cytoreduction score (CC-score) (6). The CC score was defined as follows: a CC-0 score indicates that no visible peritoneal seeding exists following the cytoreduction; a CC-1 score indicates that tumor nodules persisting after cytoreduction are <2.5 mm, which is a nodule size thought to be penetrable by intracavity chemotherapy and would, therefore, be designated as a complete cytoreduction; a CC-2 score indicates tumor nodules between 2.5 mm and 2.5 cm; and a CC-3 score indicates tumor nodules >2.5 cm or a confluence of unresectable tumor nodules at any site within the abdomen or pelvis. A bilateral pelvic and retroperitoneal lymphadenectomy was performed in case of suspicious lymph nodes on imaging or intraoperatively.

### HIPEC

HIPEC was performed with cisplatin 75 mg/m^2^ for 90 min using the closed method unless there was a contraindication to the procedure. Because HIPEC requires an out-of-pocket expenditure, it was performed only for those who could afford the additional cost and consented to the procedure. It was not performed in patients who were at undue risk of complications in the surgeon’s opinion, such as age >70 years or age >65 years with multiple comorbidities. PIPAC, as a modality of delivering intraperitoneal chemotherapy is being increasingly explored in peritoneal carcinomatosis. Although none of our patients received PIPAC, its role in the NACT setting for locally advanced ovarian cancer is worth considering and perhaps future prospective studies may provide an answer to its value addition in the treatment of locally advanced ovarian cancer.

### Pathological evaluation

The pathological evaluation was performed using a previously defined protocol for peritonectomy specimens and based on the existing guidelines for the ovarian primary and regional nodes (19, 20). The peritoneal cavity was divided into three regions: the upper region comprising of regions 1, 2, and 3 of Sugarbakers PCI, the middle region comprising of regions 0, 4, and 8, and the lower region comprising of regions 5, 6, and 7. The omentum was evaluated separately from the structures in region 0 and each small bowel region (9–12) as well. The PeRitOneal MalIgnancy Stage Evaluation online application (e-PROMISE) was used to define anatomical structures in each region of the peritoneal cancer index (21). The pathological PCI was calculated based on the size and distribution of tumor nodules on histopathological evaluation and was recorded systematically for each peritoneal region and compared with surgical PCI. The pathological response to chemotherapy was graded based on the chemotherapy response score developed by Bohm et al. (22). BRCA mutation testing was not performed for all patients.

### Evaluation of morbidity

The 90-day morbidity and mortality rates were recorded. The Clavein–Dindo classification was used to determine the morbidity. Grades 3 and 4 were considered major morbidity.

### Adjuvant chemotherapy and maintenance therapies

Adjuvant chemotherapy was started within 4–6 weeks of surgery and continued for up to six cycles. For patients who received all six cycles before surgery, an additional two to three cycles were administered at the discretion of the treating oncologist. Maintenance therapy with bevacizumab or PARP inhibitors was used at the oncologist’s discretion.

### Follow-up

Routine 3-monthly follow-up includes clinical exams, CA-125 dosages, and cross-sectional imaging studies as deemed suitable for the first two years, and every 6 months thereafter. The diagnosis of recurrence is made according to the Gynecologic Cancer Inter Group (GCIC) criteria. Recurrence within 6 months (platinum resistant recurrence) and within 12 months (early recurrence) of completing the last dose of platinum-based chemotherapy was recorded.

### Statistical analysis

Categorical data were described as number (%). Abnormally distributed continuous data were expressed as the median and range. Categorical data were compared with the chi-square test. For comparison of median values, the independent sample t-test was used and for means, the Mann–Whitney U test. A p-value of <0.05 was considered statistically significant. Receiver operating curves (ROC) were applied to determine the best possible score of pathological (pPCI) in predicting survival. We used the Youden’s index (23) to determine the threshold value that best defined the pPCI value on the ROC curve. The correlation between KELIM and pPCI was tested using Pearson’s correlation. The prognostic factors that were evaluated were the surgical and pathological PCI, number of NACT cycles, CC-score, HIPEC, chemotherapy response grade (the term is used instead of chemotherapy response score to avoid confusion with CRS), KELIM score and grade 3–4 complications rates.

Survival curves were calculated using the Kaplan–Meier test and factors affecting survival were compared using the log-rank test for univariate analysis and the Cox proportional hazard model for multivariable analysis. A p-value of <0.05 was considered statistically significant. Survival was calculated from the date of CRS and HIPEC. SPSS version 27 was used for all statistical calculations.

## Results

From January 2018 to June 2023, 171 patients undergoing interval CRS with or without HIPEC and having a minimum follow-up of 6 months from the last dose of platinum-based chemotherapy were included. All patients had high grade serous carcinoma of the ovary, fallopian tube or that arising from the peritoneum. A total of 101 patients (66%) received three to four cycles of NACT and 52 (34%) received more than four cycles. The median Surgical PCI was 12 [range: 4–26]. A CC-0 resection was obtained in 149 patients (87.7%) and CC-1 in 17 (9.9%) patients. The KELIM score was less than one in 53 patients (31%) and greater than one in 118 patients (69%). HIPEC was performed in 67% of patients (**Table 1**). The 90-day major morbidity was 31 patients (18.1%) and three patients (1.9%) died within 90 days of surgery. The details of the complications are provided in **Table 1**.

**Table 1:** Clinico pathological characteristics of 171 patients treated with CRS +/-HIPEC.

Variables	All Patients n=171 N (%)
Median Age [Range]	46 [18-78]
Median Chemotherapy Cycles [Range]	4 [3-6]
**HIPEC** Yes **No**	115(67.2%) 56 (32.7%)
**No. Of Organs Resected** Less than 3 Greater than 3	242(64.6%) 132 (35.4%)
CC Score * CC0 CC1 CC2/CC3	149(87.2%) 17(9.9%) 5 (2.9%)
**KELIM score** **< than 1** **>than 1**	53(31%) 118(69%)
**Surgical PCI (sPCI)(Median)**	12(4-26)
**sPCI>pPCI** **sPCI=pPCI** **sPCI<pPCI**	73(42%) 30(17.5%) 68(39%)
**Pathological PCI (pPCI)(Median)**	8.7 (0-26)
**Pathological PCI #** <8 >8	92 (53.8) 79(46.2)
**Chemo Response Grade** **1** **2** **3**	30 (17.5) 116 (69) 25 (14.6)
**Platinum Resistant disease** **Yes** **No**	14 (8.2) 157 (91.8)
Median Blood Loss [Range]	750ml[500-2000]
Median Duration of Surgery [Range]	600min[250-600]
Median Hospital Stay [Range]	18days [7-69]
Median ICU stay [Range]	3 day [0-13]
**Grade 3-4 Complications** Yes No	72 (19.3%) 302 (80.7%)
**90 day Morbidity** No Yes	140(81.9%) 31(18.1)
**Type of complications** Rectovaginal Fistula Anastomotic leak Intestinal Perforation Hemorrhage Urinary Fistula SSI Pulmonary Abdominal Abscess DVT Neutropenia	3 (0.8) 13 (3.4) 2 (0.5) 2 (0.5) 4 (1.0) 8 (2.1) 22 (5.8) 6 (1.6) 2 (0.5) 10 (2.6)
**BRCA Mutation** **Yes** **Not Done** **HRD tested** **HRD Mutated**	20 151 20 2
**Maintainence Bevacizumab** **Yes** **No**	44 127

### Pathological findings

The median pathological PCI was 8.7 [range: 0–26] (**Table 1** **).** A complete pathological response to NACT was observed in 25 patients (14%). The concordance between the sPCI and pPCI was seen in 17.5% of patients.

#### Statistical correlation between KELIM and path PCI and ROC curves

**Figure 1:**
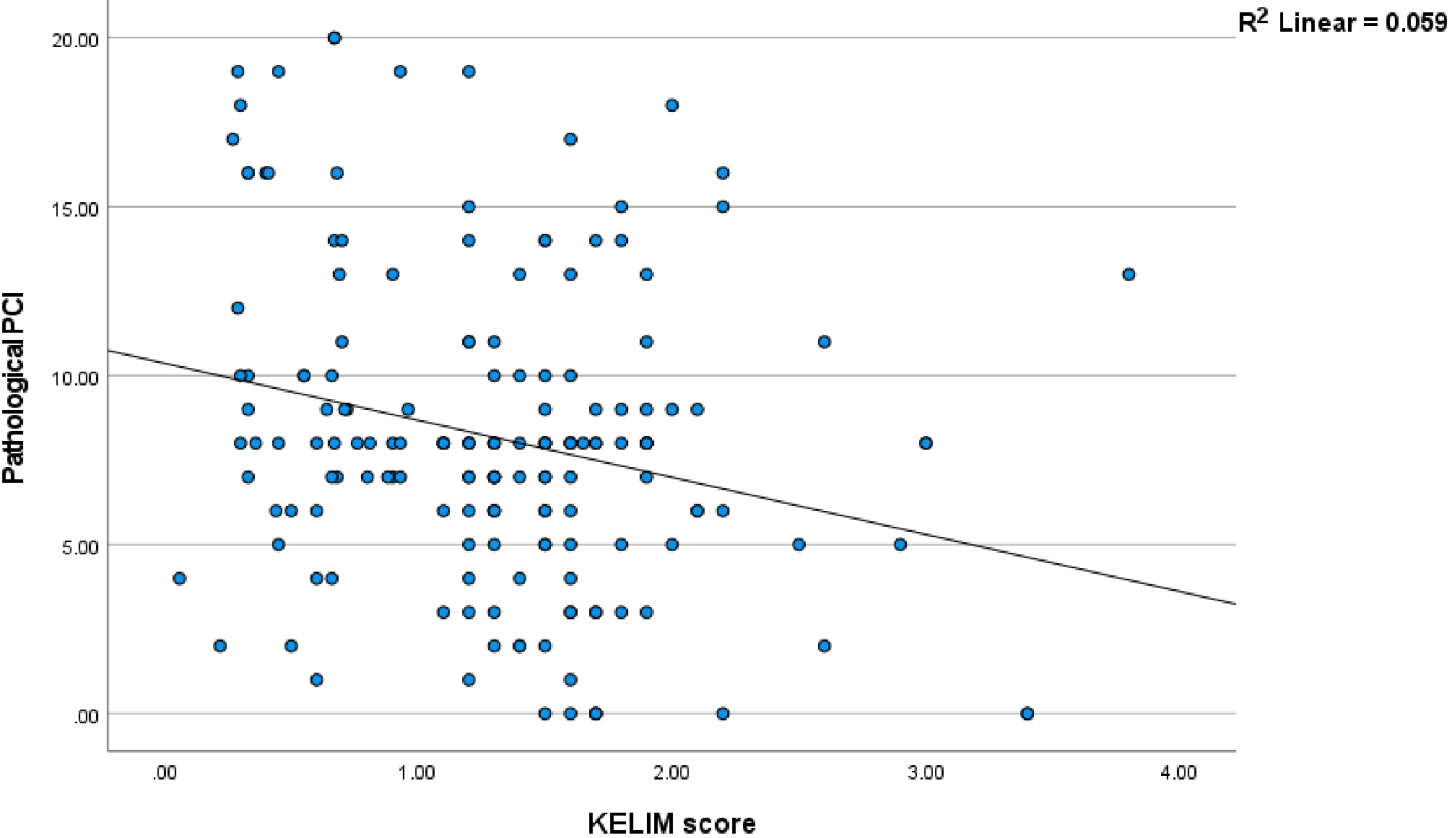
Correlation graph: Pathological PCI and KELIM score.

**Figure 2:**
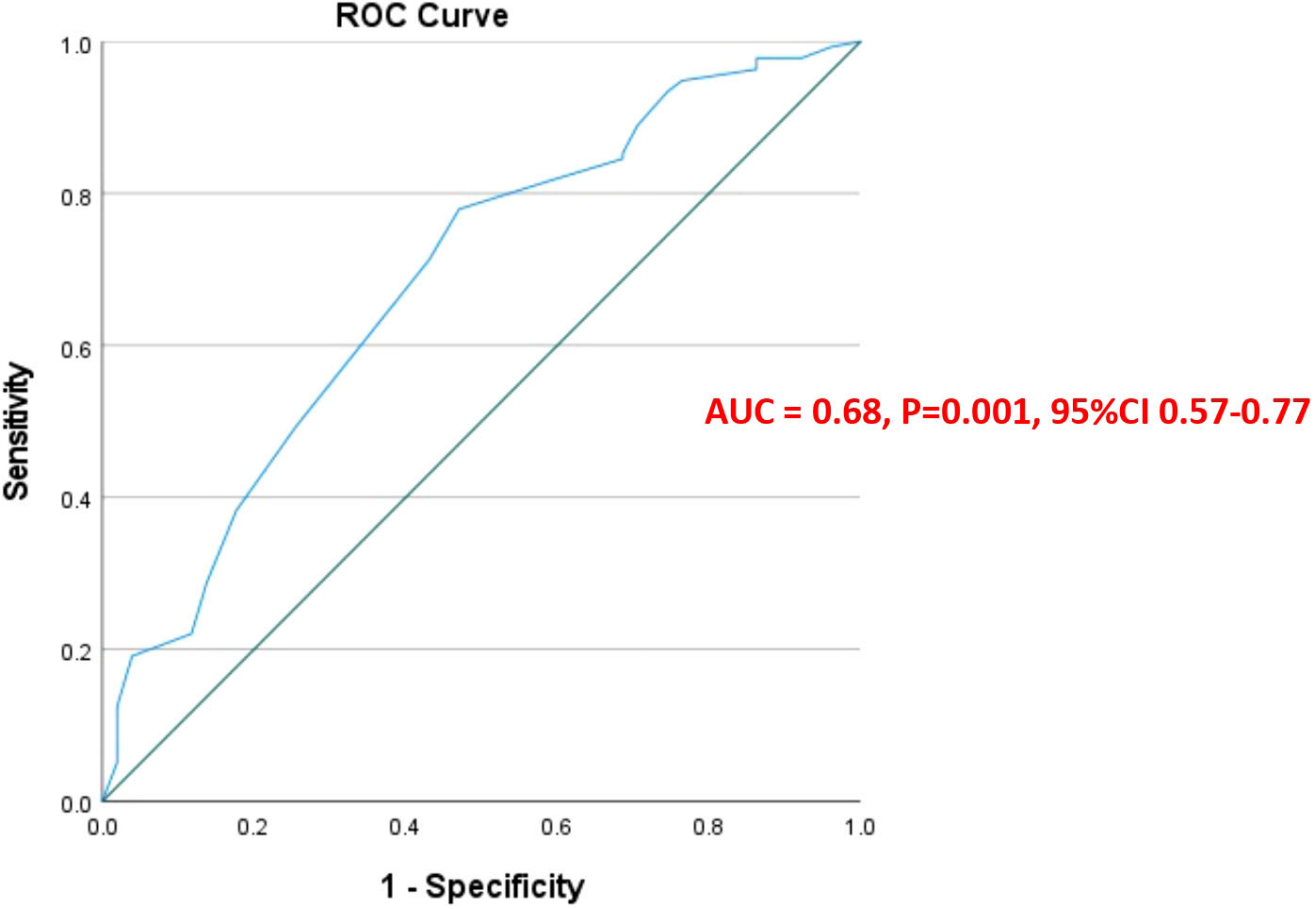
ROC curve: Pathological PCI.

There was a statistically significant linear negative correlation between the KELIM score and pathological PCI (r = −0.24 p <0.001) [95%CI (−.0.38 to −0.97)] (**Figure 1**). To explain this further, as the KELIM Score increased, the pathological PCI decreased. To determine the best possible value of pathological PCI score as a prognostic marker for survival, ROC curves were applied. The AUC for pathological PCI was [0.68, p = 0.001, CI (0.60–0.77)], for 5-year survival. From the ROC curves, a pathological PCI value of 8 was taken as the reference cut off value with a sensitivity of 82% and specificity of 67% survival (**Figure 2**).

#### Early recurrence and survival

**Figure 3:**
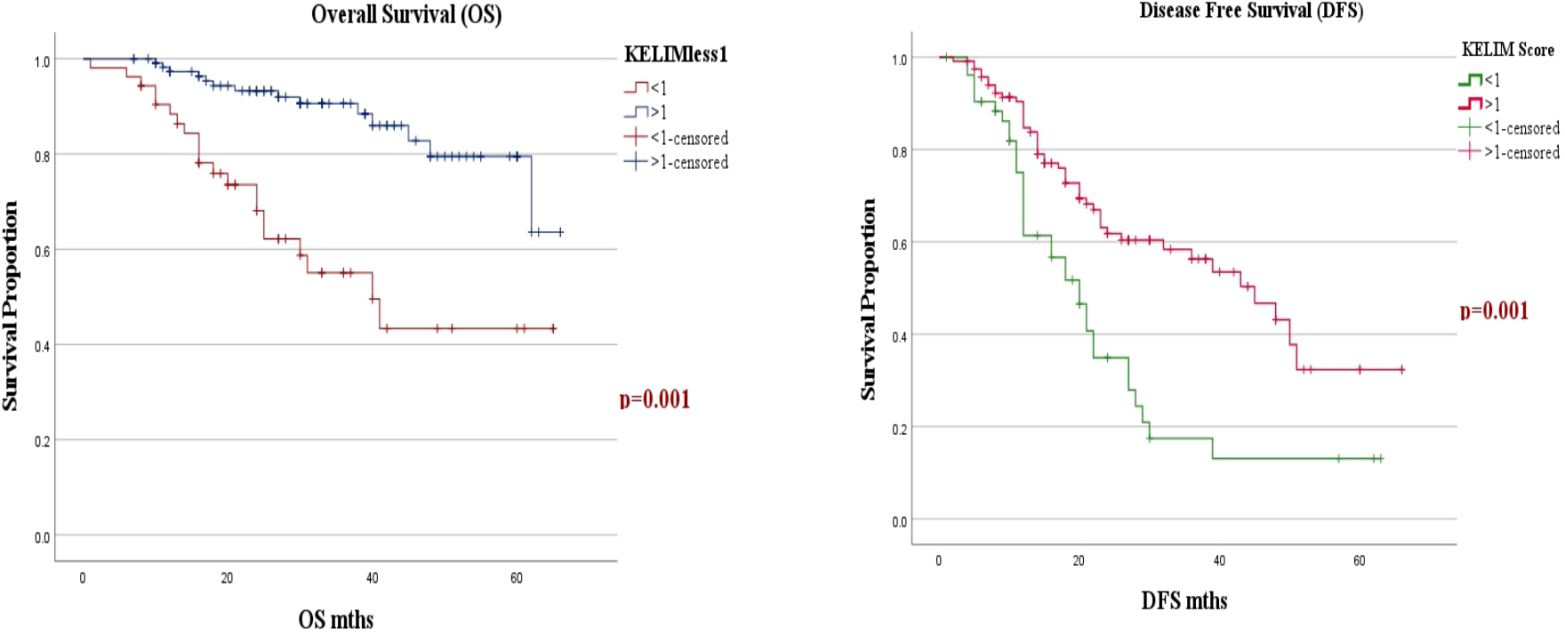
**(A, B)** Kaplan–Meier Curve for 5-year overall survival and disease-free survival, separated by KELIM score.

**Figure 4:**
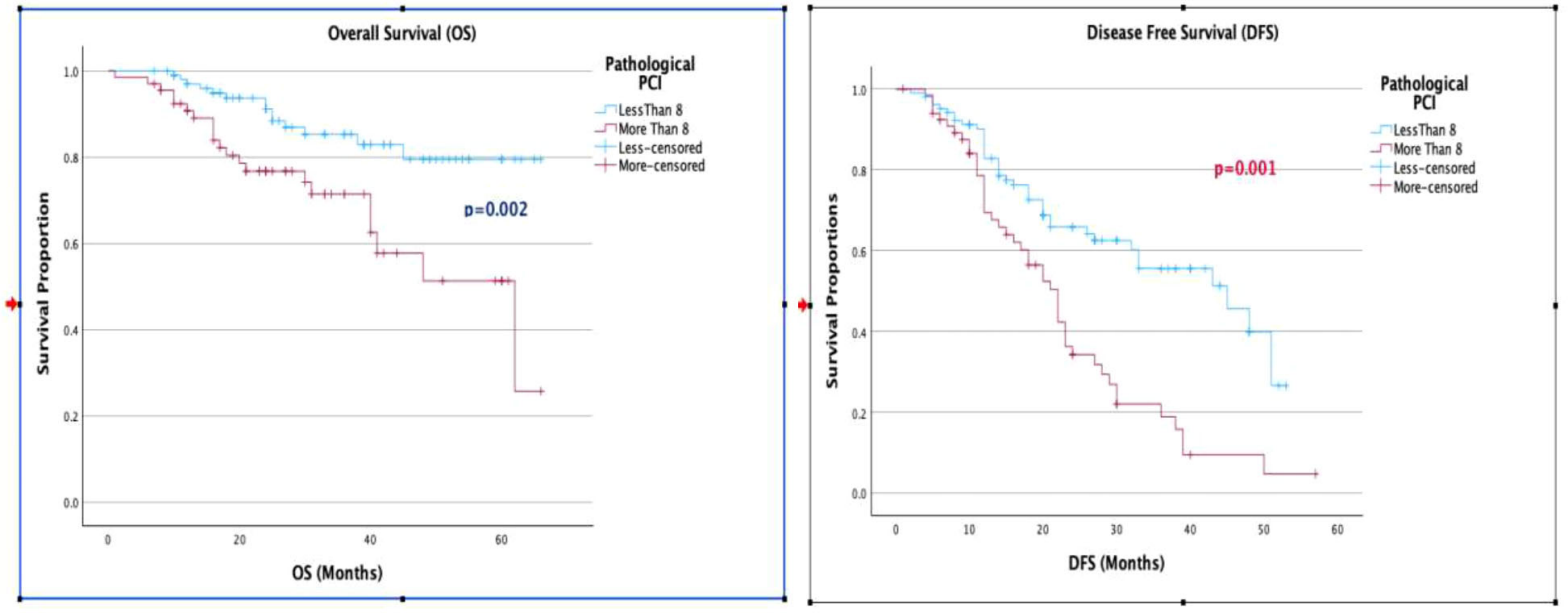
**(A, B)** Kaplan-Meier curve for 5-year overall survival and disease-free survival, separated by pathological PCI.

At a median follow-up of 33 months (range: 1–66 months), 81 patients (47.4%) developed recurrence or disease progression. Platinum-resistant recurrence (PRR) was observed in 14 patients (8.2%), and a lower KELIM score (<1) was associated with platinum-resistant recurrence (p = 0.036). The median OS of the entire cohort was not reached (NR) and median DFS was 27 months (**Figures 3**, **4**).

When evaluating the factors affecting OS and DFS, a KELIM score greater than 1 was associated with improved OS and DFS in both univariate and multivariate analyses. The median OS for a KELIM score <1 was 40 months, compared to not reached for a KELIM score >1 (p = 0.001). The median DFS was 20 months for a KELIM score <1, compared to 40 months for a KELIM score >1 (p = 0.001). Additionally, a pathological PCI with a cut-off value of 8 showed improved OS (p = 0.02) and DFS (p = 0.001), in both univariate and multivariate analyses (**Table 2**, **Figures 3**, **4**).

**Table 2:** Factors affecting overall survival and progression free survival.

Variable	Overall Survival			Disease Free Survival		
	Univariate Analysis	Multivariate analysis		Univariate Analysis	Multivariate Analysis	
	P-value	P-value	HR (95%CI)	P-value	P-value	HR (95%CI)
Complete Cytoreduction (CC0/1 vs CC2/3)	0.61			0.4		
**Path PCI** **<8 vs >8**	0.03	0.02	1.1 (0.66–0.88)	**0.001**	**0.04**	**1.2 (0.63–0.89)**
HIPEC vs No HIPEC	0.48			0.94		
**KELIM score** >1 vs <1	**0.001**	**0.001**	**2.1 (0.47–0.87)**	0.001	0.03	1.1 (0.6–0.94)
CRG score	0.56			0.03		
**No. of Organs resected** Less than 3 vs Greater Than 3	0.33			0.14		
**Complications** **Yes vs no**	0.06			0.14		

*Statistical analysis, Kaplan–Meir curve, Log-rank test for univariate analysis. variables, Multivariable Analysis, Cox Proportional hazard model.

Bold Values indicate Statistically significant values.

## Discussion

Complete cytoreduction, where no “gross visible disease” remains, is the mainstay of management for locally advanced epithelial ovarian cancer in both the primary and interval setting. Therefore, the volume of the disease, which is determined intraoperatively at the time of surgery, is the most important prognostic factor for patients undergoing cytoreductive surgery (3–5). In the interval setting, after neoadjuvant chemotherapy (NACT), however, this intraoperative PCI assessment is subjective and may be overestimated or underestimated (12, 24). Studies have evaluated the cut-off value of PCI to predict the completeness of cytoreduction after NACT, and this value was set at 17 (25). Another study determined a surgical PCI score of 13 (24) as the cut-off to assess the extent of disease in advanced serous EOC patients and proposed that it may help in predicting complete surgical cytoreduction but did not qualify as a predictor of survival.

However, there are no studies in the literature that have evaluated the best possible value of pathological PCI that can serve as a prognostic marker for survival following cytoreductive surgery after NACT.

In our study, we applied ROC curves to determine the best possible value of the pathological PCI score as a prognostic marker for survival in patients with stage IIIC high-grade serous epithelial ovarian cancer treated with neoadjuvant chemotherapy and interval cytoreductive surgery, with or without HIPEC. From the ROC curves, a pathological PCI value of 8 was taken as the reference cut off value with a sensitivity of 82% and specificity of 67% for 5-year survival. This defined pPCI cut-off of 8 was further evaluated as a factor affecting OS and DFS and was found to improve OS and DFS in both univariate and multivariate analysis.

To our knowledge, there are only a few studies in the literature that investigate the role of the KELIM score in the neoadjuvant setting for the treatment of advanced ovarian cancer. The prognostic value of KELIM on OS and DFS has been established in various studies. Besides its prognostic significance, in many studies, KELIM has been identified as an indicator of chemosensitivity and is found to be associated with radiological response during neo-adjuvant chemotherapy and the likelihood of complete resection at interval cytoreductive surgery (26–28). Two recent meta-analyses (13, 29) that studied the KELIM score in the adjuvant, neoadjuvant, and recurrent settings showed that it is an independent prognostic biomarker for progression-free and overall survival.

The study by Ducoulombier et al. (30) was a retrospective cohort of 54 patients that showed the KELIM score as an independent predictor for optimal cytoreduction after neoadjuvant chemotherapy. Most of these studies have used KELIM as a predictive factor for achieving optimal cytoreduction. The higher the KELIM score, the greater the chemosensitivity, and therefore, the higher the probability for optimal cytoreduction.

However, the correlation between chemosensitivity and the amount of residual disease after interval cytoreductive surgery has not been evaluated in any study. In our study there was a strong correlation between the KELIM score and pathological PCI. Higher KELIM scores correlated with lower pathological PCI, indicating that increased chemosensitivity was associated with reduced disease burden at surgery, which was objectively confirmed in pathological evaluation. In all our patients, regardless of the KELIM score, more than 80% achieved a CC0. Furthermore, in our study, on univariate and multivariate analyses, a KELIM score <1 and PCI >8 were associated with the worst outcome.

This study and its further validation in different cohorts is important because, in ovarian cancer, especially after NACT, there are currently no established prognostic factors. Identifying prognostic and predictive factors is important as they may be useful in making therapeutic decisions such as planning adjuvant therapy, changing therapeutic strategies or implementing maintenance therapy with targeted agents.

To explain this further, currently, maintenance chemotherapy, e.g., bevacizumab is offered to patients with incomplete cytoreduction after primary cytoreductive surgery (31, 32) There is still no clarity regarding the appropriate approach for maintenance therapy for patients who have undergone a CC0 surgical cytoreduction in the interval setting. Furthermore, in the real-world setting, these surrogate markers could be useful tools to guide clinicians for further treatment, especially in situations where HRD testing/molecular testing is unfunded and in resource-driven settings. Therefore, such patients with a low KELIM or high pPCI >8, despite a CCO may warrant different therapeutic agents or maintenance therapy.

Lastly, our study is not without its limitations. Very few of our patients received maintenance therapy with bevacizumab or PARP inhibitors. This was because of two reasons: the use of these maintenance therapies was at the discretion of the treating medical oncologist, and ours is a resource-driven setup, and not all patients have the financial capacity to afford maintenance therapy. Therefore the impact of these therapeutic agents on survival in our cohort of patients could not be evaluated.

To the best of our knowledge ours is the only study to evaluate the importance of pathological PCI and not only show its significance as a prognostic marker of survival but also determine the best cut-off value that could impact survival outcomes. Despite the limitations, our findings merit consideration and further evaluation in prospective studies with a larger patient sample size.

## Conclusion

Following interval cytoreductive surgery, despite optimal complete cytoreductive surgery, a pathological PCI of 8 and above, and a KELIM score less than 1 are poor prognostic indicators of overall survival and disease-free survival. The KELIM score has a strong negative correlation with the amount of residual disease post-cytoreductive surgery, as determined by the pPCI. However these results need to be validated in further studies.

## Data Availability

The raw data supporting the conclusions of this article will be made available by the authors, without undue reservation.
